# Creating a quality improvement culture in standardized/simulated patient methodology: the role of professional societies

**DOI:** 10.1186/s41077-017-0051-4

**Published:** 2017-10-03

**Authors:** Debra Nestel, Jan Roche, Alexis Battista

**Affiliations:** 1 Department of Surgery, University of Melbourne, Melbourne, Australia; 2 Chameleon Simulation Centre, School of Medicine and Public Health, Faculty of Health and Medicine, Newcastle University, Newcastle, Australia; 3 Graduate Programs in Health Professions Education, Department of Medicine, F. Edward Hébert School of Medicine, Uniformed Services University of the Health Sciences, Bethesda, USA; 4 Monash Institute for Health and Clinical Education, Faculty of Medicine, Nursing & Health Sciences, Monash University, Clayton, Victoria 3168 Australia

Contemporary professional communities are

required to adhere to high ethical standards and uphold themselves to, and are accepted by, the public as possessing special knowledge and skills in a widely recognised, organised body of learning derived from education and training at a high level, and who are prepared to exercise this knowledge and these skills in the interests of others [1].

Standards apply in many healthcare fields as a means of promoting the provision of safe, evidenced-based practice *and* nurturing a quality improvement culture.

Healthcare simulation enables users, or systems, to explore, reflect, and review practice, provided the design of the simulation is rigorous and appropriate safety measures are in place [2]. As the industry matures, the technology and procedures used to create authentic simulated experiences are also developing in parallel with realization of the need for standards and guiding professional bodies [1]. In healthcare simulation, some examples of professionalization include:

- Growth of societies and networks addressing the needs of simulation practitioners. These societies can be characterized as being local, regional, national, or international; or by different foci, such as clinical orientation (e.g., Pediatrics—International Pediatric Simulation Society—http://ipssglobal.org/), professional discipline (e.g., Nursing—International Nursing Association for Clinical Simulation and Learning (INACSL)—https://www.inacsl.org/i4a/pages/index.cfm?pageid=1), and simulation modality (e.g., Standardized patients—Association of Standardized Patient Educators (ASPE)—http://www.aspeducators.org/).
- Development of standards for simulation practitioners (e.g., Certification—Society for Simulation in Healthcare—http://www.ssih.org/Certification).
- Development of standards for simulation programs or centers (e.g., SSH—http://www.ssih.org/Accreditation, ASPIRE at Association for Medical Education in Europe—https://amee.org/amee-initiatives/aspire#about-aspire), Society in Europe for Simulation Applied to Medicine—https://www.sesam-web.org/accreditation/, Royal College of Physicians and Surgeons of Canada—http://www.royalcollege.ca/rcsite/cpd/accreditation-simulation-programs-e, and the American College of Surgeons—http://bulletin.facs.org/2014/07/the-acs-accredited-education-institutes-fellowship-program-training-leaders-in-simulation-based-education/#The_AEI_Program.
- Growth of award or postgraduate degree courses in healthcare simulation or development of subjects on important components of simulation in clinical/health professional educator and/or technology programs.
- Evolving literature on healthcare simulation practices reporting research and reference books documenting educational approaches.

With professionalization comes a responsibility for organization and progress. *Advances in Simulation* has published the ASPE Standards of Best Practice (SOBP) [3]. These standards are designed for standardized/simulated patient (SP) educators who work with simulated participants, defined as individuals who are trained to portray patients and others involved in healthcare settings. Simulated participant methodology is a specialized practice in healthcare simulation that has at its core, the promotion and support of simulated participants.

The Association of Standardized Patient Educators (ASPE) describes itself as a global organization whose mission is to share advances in SP-based pedagogy, assessment, research, and scholarship as well as support the professional development of its members [4]. The SOBP, which are intended to be used in conjunction with the International Nursing Association for Clinical Simulation and Learning Standards of Best Practice: Simulation^SM^ [5], have five underpinning values, including safety, quality, professionalism, accountability, and collaboration [3]. The standards further articulate five domains of best practice, including:

1. Safe work environment
2. Case development
3. SP training for role portrayal, feedback, and completion of assessment instruments
4. Program management
5. Professional development [3]

The SOBP are directed at *SP educators*, rather than simulated participants, and are intended to provide guidance as essential and sometimes aspirational practices to optimize the conditions for SPs to work effectively and safely. Importantly, the SOBP acknowledge the complexity of simulation with their focus on a single modality and of special importance is that the “modality” comprises live humans. Although SP methodology has wide-ranging benefits for training, it is vital to be aware of the potential risks to SPs, including psychological and physical harm. This is particularly important because the unintended devaluing of simulated participants remains in common parlance when referring to “the use of simulated patients.” Nestel et al. (2011) have proposed reconsidering this phrase to the more positive “working with SPs.” Rather than regarding SPs as tools or objects of use, this small but significant statement emphasizes the crucial contributions that simulated participants make in supporting learning.

In addition, the SOBP statements acknowledge that context is critical for defining competence [6] and, thus, are intended to have sufficient flexibility to be broadly relevant. The SOBP are also framed as a “living document” acknowledging the importance of changes in competence and practice over time. It is foreseeable that some standards deemed appropriate today will be inappropriate or else defined differently a decade from now. For example, although the SOBP acknowledge the roles that real patients may play, including that of a stakeholder (Domain 1) or subject matter expert (Domain 4), real patients are otherwise not mentioned in the SOBP. This may be one area that evolves as real and simulated patients and SP educators work more collaboratively. Another area of collaboration could be between an SP educator and an SP in the writing of learning objectives as reported in the work of Snow [7]. Moreover, the SOBP largely frame feedback as a one-way process of supporting SPs in giving feedback (Domain 3). Conversational feedback is likely to grow in importance [8, 9]. Finally, SPs themselves do not seem to have had an active and direct role in the development process.

Elsewhere, it has been argued that simulated participants may not exist in the future [10] as sophisticated screen-based, emotionally expressive, virtual patients will take on these roles. The blurring between real worlds and manufactured realities could lead to this conclusion. However, SPs remain the physical and psychological embodiment of a real patient affording the learner the opportunity to attend to physical examinations and to deal with sensitive and complex communication simultaneously [11]. ASPE will not only have an important role in optimizing SP contributions to the professional development of healthcare professionals, but also in maintaining their prominence and in ensuring SP safety. The interface between SPs and technology may have exciting new facets of practice for SP educators.

*Advances in Simulation* publishes work that reports all simulation modalities and have published two articles with simulated participants as the focus. Both articles were reviews and targeted different facets of SP methodology—the role of SPs in facilitating the development of clinical competence [12] and considerations of children and adolescents as SPs [13]. A future initiative of *Advances in Simulation* will be the tagging of all SP-based articles enabling easy searching for SP educators and others interested in the practice.

As the health sector is increasingly burdened by heavy and complex workloads, the skills and knowledge of clinicians need to be constantly honed and updated. Simulation offers the chance to practice all facets of healthcare from the simple to complex and both common and rare events without consequences to real patients by learning with SPs. Professionalization is the impetus for these standards. Practitioners have come together to articulate standards that will shape the expectations of their community’s work. These aspirational standards will facilitate the highest quality SP practices for integration of physical and communication skills in a safe and respectful environment. We applaud the work of ASPE in the development of these SOBP in the promotion of a quality improvement culture that will go some way to ensure the growth, integrity, and safe application of SP-based educational endeavors [3].
